# Increasing the effectiveness of psychotherapy in routine care through blended therapy with transdiagnostic online modules (PsyTOM): study protocol for a randomized controlled trial

**DOI:** 10.1186/s13063-022-06757-0

**Published:** 2022-09-30

**Authors:** Carmen Schaeuffele, Manuel Heinrich, Solveig Behr, Friederike Fenski, Leona Hammelrath, Pavle Zagorscak, Alessa Jansen, Steffi Pohl, Johanna Boettcher, Christine Knaevelsrud

**Affiliations:** 1 Department of Education and Psychology, Freie Universität Berlin, Berlin, Germany; 2 Department of Clinical Psychology and Psychotherapy, Psychologische Hochschule Berlin, Berlin, Germany; 3 Bundespsychotherapeutenkammer, Berlin, Germany

**Keywords:** Blended care, Blended therapy, Routine care, Transdiagnostic, Internet-based, Approach-bridging, Randomized controlled trial

## Abstract

**Background:**

In blended therapy, face-to-face psychotherapy and Internet-based interventions are combined. Blended therapy may be advantageous for patients and psychotherapists. However, most blended interventions focus on cognitive behavioral therapy or single disorders, making them less suitable for routine care settings.

**Methods:**

In a randomized controlled trial, we will compare blended therapy and face-to-face therapy in routine care. We intend to randomize 1152 patients nested in 231 psychotherapists in a 1:1 ratio. Patients in the blended therapy group will receive access to a **t**herapeutic **on**line **i**ntervention (TONI). TONI contains 12 transdiagnostic online modules suited for psychodynamic, cognitive behavioral, and systemic therapy. Psychotherapists decide which modules to assign and how to integrate TONI components into the psychotherapeutic process to tailor treatment to their patients’ specific needs. We will assess patients at baseline, 6 weeks, 12 weeks, and 6 months. Patients enrolled early in the trial will also complete assessments at 12 months. The primary outcomes are depression and anxiety at 6-month post-randomization, as measured by PHQ-8 and GAD-7. The secondary outcomes include satisfaction with life, level of functioning, personality traits and functioning, eating pathology, sexual problems, alcohol/drug use, satisfaction with treatment, negative effects, and mental health care utilization. In addition, we will collect several potential moderators and mediators, including therapeutic alliance, agency, and self-efficacy. Psychotherapists will also report on changes in symptom severity and therapeutic alliance. Qualitative interviews with psychotherapists and patients will shed light on the barriers and benefits of the blended intervention. Furthermore, we will assess significant others of enrolled patients in a sub-study.

**Discussion:**

The integration of online modules which use a common therapeutic language and address therapeutic principles shared across therapeutic approaches into regular psychotherapy has the potential to improve the effectiveness of psychotherapy and transfer it into everyday life as well help save therapists’ resources and close treatment gaps. A modular and transdiagnostic setup of the blended intervention also enables psychotherapists to tailor their treatment optimally to the needs of their patients.

**Trial registration:**

German Clinical Trials Register (DRKS) DRKS00028536. Registered on 07.06.2022.

## Administrative information

Note: The numbers in curly brackets in this protocol refer to the SPIRIT checklist item numbers. The order of the items has been modified to group similar items (see http://www.equator-network.org/reporting-guidelines/spirit-2013-statement-defining-standard-protocol-items-for-clinical-trials/).

**Table:** 

Title {1}	Increasing the effectiveness of routine psychotherapy through blended therapy with transdiagnostic online modules (PsyTOM): study protocol for a randomized controlled trial
Trial registration {2a and 2b}.	The trial is registered at the German clinical trials registry DRKS: DRKS00028536. The trial was registered on 07.06.2022.
Protocol version {3}	2022–05-30, version 1
Funding {4}	This study is funded by the Innovation Committee (Innovationsausschuss) of the Joint Federal Committee (Gemeinsamer Bundesausschuss, GBA, no: 01VSF19041).
Author details {5a}	Carmen Schaeuffele, PhD, Department of Education and Psychology, Freie Universität Berlin, Berlin, Germany, carmen.schaeuffele@fu-berlin.de (corresponding author) Manuel Heinrich, MSc, Department of Education and Psychology, Freie Universität Berlin, Berlin, Germany, Solveig Behr, MSc, Department of Education and Psychology, Freie Universität Berlin, Berlin, Germany, s.behr@fu-berlin.de Friederike Fenski, MSc, Department of Clinical Psychology and Psychotherapy, Psychologische Hochschule Berlin, Berlin, Germany, f.fenski@tp.phb.de Leona Hammelrath, MSc, Department of Education and Psychology, Freie Universität Berlin, Berlin, Germany, leona.hammelrath@fu-berlin.de Pavle Zagorscak, PhD, Department of Education and Psychology, Freie Universität Berlin, Berlin, Germany, zagorscak@zedat.fu-berlin.de Alessa Jansen, PhD, Bundespsychotherapeutenkammer, jansen@bptk.de Steffi Pohl, PhD, Department of Education and Psychology, Freie Universität Berlin, Berlin, Germany, steffi.pohl@fu-berlin.de Johanna Boettcher, PhD, Department of Clinical Psychology and Psychotherapy, Psychologische Hochschule Berlin, Berlin, Germany, johanna.boettcher@phb.de (shared last authorship) Christine Knaevelsrud, PhD, Department of Education and Psychology, Freie Universität Berlin, Berlin, Germany, christine.knaevelsrud@fu-berlin.de (shared last authorship)
Name and contact information for the trial sponsor {5b}	Innovationsausschuss beim Gemeinsamen Bundesausschuss, Postfach 12 06 06, 10,596 Berlin, info@if.g-ba.de
Role of sponsor {5c}	No sponsor has a part in the study design, data collection, management, analysis and interpretation, writing of the report, or decision to submit the report.

## Introduction

### Background and rationale {6a}

Over the course of 12 months, about one-third of Germans meet the criteria for at least one mental disorder—of which nearly 50% have more than one mental disorder [1]. High prevalence and comorbidity rates offset significant treatment gaps and waiting times to access psychotherapy [2, 3]: Less than 25% of patients seek treatment, and if they do, they are faced with waiting times of about 5 months before starting psychotherapy in German outpatient routine care.

Blended therapy may constitute a promising way to close treatment gaps. In blended therapy, Internet-based interventions and face-to-face psychotherapy are combined in different ways [4, 5]. Internet-based interventions can serve as an add-on to psychotherapy without being integrated into face-to-face therapy [5]. Internet-based interventions can also be an integral part of face-to-face therapy. For example, topics and exercises are discussed in in-person sessions, and patients then continue to work on these topics online. The results from a systematic review suggest that blended therapy may save clinician time, lower dropout rates, and maintain changes achieved during psychotherapy [5]. However, while the effects of blended treatments are promising, the overarching potential of blended therapy is difficult to determine since blended treatments encompass such a wide array of different setups [5]. In addition, direct comparisons between face-to-face and blended therapy in routine care are still sparse. Those studies that compared both treatment formats directly yielded small to medium between-group effects in favor of blended therapy [6]. Blended treatments combine the benefits of Internet-based and in-person treatments: On the one hand, they provide the flexibility and potential to integrate psychotherapy into the everyday lives of patients inherent to stand-alone Internet-based interventions. Additionally, as part of the treatment process may be conducted via online self-help materials, blended interventions have the potential to save clinician time. On the other hand, they offer the personal contact of in-person psychotherapy that may capitalize on in-session processes and respond to crises more adequately [7, 8].

So far, blended therapy is not implemented in outpatient routine care in Germany. Several factors constitute barriers to the implementation of blended therapy. First and foremost, the needs of practicing psychotherapists and their patients and existing blended interventions are not aligned: The majority of Internet-based interventions focus on cognitive behavioral therapy (CBT) [9, 10], target anxiety and depression [11], and allow for little flexibility and tailoring.

The strong CBT focus does not reflect the therapeutic orientation of psychotherapists practicing in routine care in Germany: More than 40% of psychotherapists have a different therapeutic orientation than CBT. The emergence of psychodynamic Internet-based interventions also underlines that other therapeutic principles besides CBT can successfully be delivered over the Internet [12]. However, instead of developing interventions specific to one therapeutic approach, it may be plausible to develop and deliver Internet-based interventions in an integrative manner that uses a common therapeutic language. This would ensure the uptake of Internet-based intervention by a wider range of psychotherapists in routine care. Besides, many psychotherapists integrate elements across different approaches of therapy, the psychotherapeutic practice of different approaches is not as distinguishable as one might expect [13–15], and different approaches share the same mechanisms of change [16, 17].

The focus on anxiety and depression neglects other prevalent mental disorders, such as the remainder of the internalizing (e.g., eating disorders and sexual dysfunctions), the whole externalizing spectrum (e.g., substance-related disorders), and thought disorders [18]. In addition, Internet-based interventions integrated into outpatient routine care need to target comorbidity appropriately, as those patients who use mental health services are typically those with more than one diagnosis [3]. For psychotherapists to embrace and integrate Internet-based interventions into their clinical practice, interventions need to be customizable and flexible. Psychotherapists emphasize that they need to be able to select and individualize treatment elements and choose how to integrate Internet-based interventions into their practice [19].

A transdiagnostic and modular blended intervention that uses a common therapeutic language has the potential to overcome these barriers. Through means of a formative research process with psychotherapists and patients, we developed existing transdiagnostic online modules into such a blended intervention using a common therapeutic language. Therapeutic online intervention (TONI) provides psychotherapists with online modules that they can integrate into their treatment to flexibly blend online components and face-to-face sessions. The aim of the current study is to investigate the effectiveness of blending TONI and face-to-face psychotherapy in routine care in Germany. For this purpose, blended psychotherapy is compared to regular face-to-face psychotherapy in routine care.

### Objectives {7}

This randomized controlled trial (RCT) evaluates whether providing psychotherapists access to online modules that they can integrate into psychotherapy benefits patients and psychotherapists. We hypothesize that patients in the blended therapy condition show larger reductions in symptoms over time than participants that solely receive face-to-face therapy. We hypothesize this effect to be small. We will investigate the treatments’ effects on the level of functioning, satisfaction with life, transdiagnostic symptoms, and personality. In addition, length of treatment, satisfaction with treatment, and negative effects are considered. We will investigate which moderators and mediators are relevant for treatment effects. We will also explore how psychotherapists and patients engage with the intervention and whether the extent of usage of the intervention moderates effects. In addition, qualitative interviews with psychotherapists and patients will shed light on the barriers and facilitators of the intervention. Furthermore, we will assess significant others of enrolled patients in a sub-study.

### Trial design {8}

A parallel-group, two-arm randomized controlled superiority trial compares the effectiveness of blended therapy to face-to-face therapy in Germany with a 1:1 allocation ratio. Quantitative and qualitative data will determine the effect of enabling psychotherapists to add online elements to their face-to-face therapy in routine outpatient care. This trial protocol is reported following the SPIRIT guidelines. The study has been registered in the German clinical trial register under DRKS00028536. The ethics committee of Psychologische Hochschule Berlin (EK202121) approved the trial.

## Methods: participants, interventions, and outcomes

### Study setting {9}

The study is jointly run by Freie Universität Berlin and Psychologische Hochschule Berlin. We will recruit psychotherapists in German routine care to participate in the study. They in turn will recruit their patients at the beginning of psychotherapy. Psychotherapists as well as patients and their significant others will complete the assessments on the TONI platform. In addition, we will conduct telephone interviews with a selection of psychotherapists and patients.

### Eligibility criteria {10}

All psychotherapists, irrespective of psychotherapeutic approach, are eligible for inclusion if they (a) take part in contractual routine psychotherapy in Germany and (b) have Internet access in their practice. Their patients who (a) are over 18 years old, (b) have access to the Internet, (c) have sufficient German language skills, and (d) can read and write are eligible for inclusion. Psychotherapists use these inclusion criteria to decide whom to invite to participate in the study. In addition, psychotherapists and patients have to verify during the registration process that these inclusion criteria apply to them. Since this is a naturalistic trial in routine care, there are no exclusion criteria regarding diagnoses or symptom severity.

### Who will take informed consent? {26a}

Psychotherapists, patients, and significant others of patients will give written informed consent online.

### Additional consent provisions for collection and use of participant data and biological specimens {26b}

Participants who take part in the interviews will consent to their participation via e-mail. No biological specimens will be collected.

### Interventions

#### Explanation for the choice of comparators {6b}

Blended therapy — the integration of Internet-based interventions in face-to-face therapy — is a promising treatment option regarding clinical effectiveness and utility. Moreover, the implementation may also help to close treatment gaps in routine care, e.g., by intensifying treatment effects. For this purpose, blended therapy is compared to regular face-to-face psychotherapy — the current gold standard of mental health care. The main hypothesis is that blended therapy will demonstrate superiority to face-to-face psychotherapy in reducing symptom distress in patients in routine care.

### Intervention description {11a}

TONI is a digital toolbox comprising modules and self-tracking tools that can be used to blend Internet-based components and face-to-face psychotherapy. TONI consists of 12 transdiagnostic modules (see Table 1 for a description of the intervention components). Every module consists of two to seven chapters. Psychotherapists can individualize modules by selecting chapters and choosing which TONI components to integrate when into their treatment. Patients can also request access to modules. In addition to the modules, TONI includes several tracker tools that allow patients to track their distress, mood, activity, sleep, craving, or individual problems which they can share with their therapist. Patients can also use a diary and have access to an “emergency kit” with emergency contacts and skills to apply during a crisis. Optionally, psychotherapists can choose to communicate with their patients via the platform.

**Table 1:** Intervention components

Module	Description of major concepts and components
Development	In chapter 1, patients learn about the biopsychosocial etiological model of distress and reflect on factors that may influence and maintain their symptoms. In chapter 2, patients reflect on their own biographical history and identify possible commonalities between fluctuations in their mental health. In chapter 3, patients develop a genogram of their family history and relations to identify intergenerational patterns that may influence them in their present.
Values and goals	In chapter 1, patients reflect on their motivation for change and identify barriers to change they have encountered in the past. Chapter 2 helps patients to identify their personal values that guide them through life. In chapter 3, patients reflect on the goals they want to work towards in therapy.
Mindfulness	In chapter 1, patients are introduced to the concept and components of mindfulness. Chapter 2 offers a selection of different internal and external mindfulness-based exercises.
Getting active	In chapter 1, patients learn about the relationship between activity and mood. In chapter 2, they reflect on which activities give them fulfillment and enjoyment. In chapter 3, they work towards implementing more activities in their everyday life.
Thoughts	In chapter 1, patients learn about the relationship between thoughts and emotions. In chapter 2, they identify how their life story shaped their thoughts. They identify and challenge their beliefs. In chapter 3, patients practice mentalization and take different perspectives.
Understanding feelings	In chapter 1, patients learn about feelings and reflect on their stance towards emotions. Chapter 2 focuses on the messages behind feelings. In chapter 3, patients reflect on how their life story shapes their emotional experience.
Dealing with feelings	In chapter 1, patients learn how to tolerate emotions. In chapter 2, patients learn more about avoidance/defense mechanisms and experiment with trying out new reactions.
Self-worth and strengths	Chapter 1 introduces the concept of self-worth, and patients reflect on the standards they set for themselves. Chapter 2 focuses on how patients can treat themselves with more kindness and self-compassion. Chapter 3 focuses on establishing which strengths and resources patients have and how they can utilize them more.
Communication	Chapter 1 introduces patients to the different meanings that messages can convey. In chapter 2, patients identify their communication patterns and reflect on their relationships with others. In chapter 3, patients learn how to communicate their needs and wishes in a nonviolent way.
Body and well-being	This module contains different chapters that are relevant for healthy living: a chapter on the relationship between body and mind, eating and body image, sleep, movement, pain and bodily complaints, sexuality, and stress. All of them include psychoeducation on the topic and exercises to try out new ways of dealing with these topics.
Addictive substances and behaviors	In chapter 1, patients are introduced to the core concepts of substance- and non-substance-related use and reflect on whether they have any wishes for change in these areas. Chapter 2 encourages patients to reflect on the pros and cons of engaging in their substance use/ addictive behavior. Chapter 3 focuses on craving and how to deal with craving. Chapter 4 focuses on relapse prevention and how to deal with relapses.
Collaboration	This module is targeted at a patient’s significant other. Chapter 1 focuses on how the significant other deals with his or her own mental well-being. In chapter 2, significant others are introduced to some of the basics of therapy and emotional well-being. Chapter 3 offers exercises that patients and their significant other can work on together, e.g., acknowledging aspects they appreciate about each other or reflecting which difficulties they have mastered in the past together.

We developed TONI by means of a formative research process including psychotherapists from all recognized therapeutic approaches in Germany (psychodynamic, cognitive-behavioral, and systemic) and patients with lived experience.

### Criteria for discontinuing or modifying allocated interventions {11b}

TONI automatically logs adherence and engagement with the online modules. We will report adherence and usage metrics. Therapists decide to what extent online modules are assigned and integrated based on the course of therapy. Therapists have access to certain usage metrics which allows them to monitor their patients’ platform usage (e.g., last login more than 4 weeks ago, % of modules completed) and address potential issues in in-person sessions or via the integrated messenger. In addition, therapists can monitor symptom progression and session ratings and adjust the intervention if necessary. Patients will be invited to assessments even if they drop out of therapy or discontinue working on modules.

### Strategies to improve adherence to interventions {11c}

Psychotherapists are informed about ways to integrate TONI into therapy and sessions in video tutorials at the beginning of the trial and in a study manual that they receive via mail. To ensure that psychotherapists invite patients to participate in the study and use TONI, they will receive a monetary compensation for the tutorials, which is paid out after including three patients, and for the usage of TONI for every patient in the blended care group. The platform automatically logs engagement with TONI. This includes numbers and duration of logins, modules/chapters assigned and completed, and messages exchanged. Patients receive e-mail notifications if a new module or assessment is available to them. Both patients and therapists also have a newsfeed on their dashboard notifying and reminding them about tasks or events.

### Relevant concomitant care permitted or prohibited during the trial {11d}

n/a: There are no prohibited additional interventions specified as this is a trial conducted in routine care. We will assess medication use and mental health care utilization to determine additional mental health care utilization.

### Provisions for post-trial care {30}

n/a: There is no ancillary post-trial care or compensation. Patients are enrolled in routine psychotherapy during the study duration.

### Outcomes {12}

Patient outcome measures consist of self-reported questionnaires, which are assessed at baseline (*t*_0_), after 6 weeks (*t*_1_), after 12 weeks (*t*_2_), and after 6 months (*t*_3_). Patients who are enrolled early and are able to complete a 12-month assessment within the duration of the project will also complete a 12-month follow-up assessment (*t*_4_). Patients who receive blended therapy will also complete an assessment for every in-person session. Psychotherapists will provide sociodemographic data regarding their own person at baseline and then rate their patients and therapeutic alliance throughout treatment in both groups. All questionnaires will be presented via the platform that also hosts the intervention. See Table 2 and the “Data collection and management” section for details of measurements at all time points.

**Table 2:** Schedule of enrollment, interventions, and assessments

**Assessment/activity**		**Enrollment**	**Allocation**	**Post-allocation**				
		***-t***_**1**_	**0**	***t***_**1**_	***t***_**2**_	***t***_**3**_	***t***_**4**_	**Others**
**Enrollment**	**Eligibility screen**	X						
	**Informed consent**	X						
	**Allocation**		X					
**Interventions**	***Treatment as usual***							
	***Blended therapy***							
***Assessment of therapists***	Sociodemographic data	X						
	BFI-10	X						
	CGI-S	X		X	X	X	X	
	CGI-C			X	X	X	X	
	WAI			X	X	X		
	Description of patient problems	X						
	Expectation of outcome and dropout	X						
	Perceived similarity	X		X				
	NEQ					X		
	SUS					X		
	Usage time					X		
***Assessment of patients***	Sociodemographic data	X						
	ACE	X						
	Perceived similarity	X		X				
	Expectation of outcome and dropout	X						
	PHQ-ADS (PHQ-8 and GAD-7)	X		X	X	X	X	
	SWLS	X		X	X	X	X	
	AQoL-8D	X		X	X	X	X	
	EDE-Q8	X				X	X	
	DUDIT-C	X				X	X	
	AUDIT-C	X				X	X	
	MGH	X				X	X	
	PID5BF +	X				X	X	
	OPD-SFK	X				X	X	
	FIMPsy	X				X	X	
	NEQ					X		
	CSQ-8					X		
	Change rating					X	X	
	SUS					X		
	ASKU	x		X	X	X	X	
	MHSES	x		X	X	X	X	
	Empowerment					X		
	TAI			X	X	X		
	WAI-SR			X	X	X		
	CPPS			X	X			
	MULTI			X	X			
	SRS/ORS (only BC)							Weekly
***Assessments of significant others***	Sociodemographic data	X						
	Expectation of outcome and dropout of patient	X						
	PHQ-ADS (GAD-7 and PHQ-8)	x				x		
	Rating of distress of patient (GAD-7 and PHQ-8)	x				x		
	IEQ	x				x		
	Therapy-associated changes					x		
	NEQ					x		

#### Participant timeline {13}

**Fig. 1:**
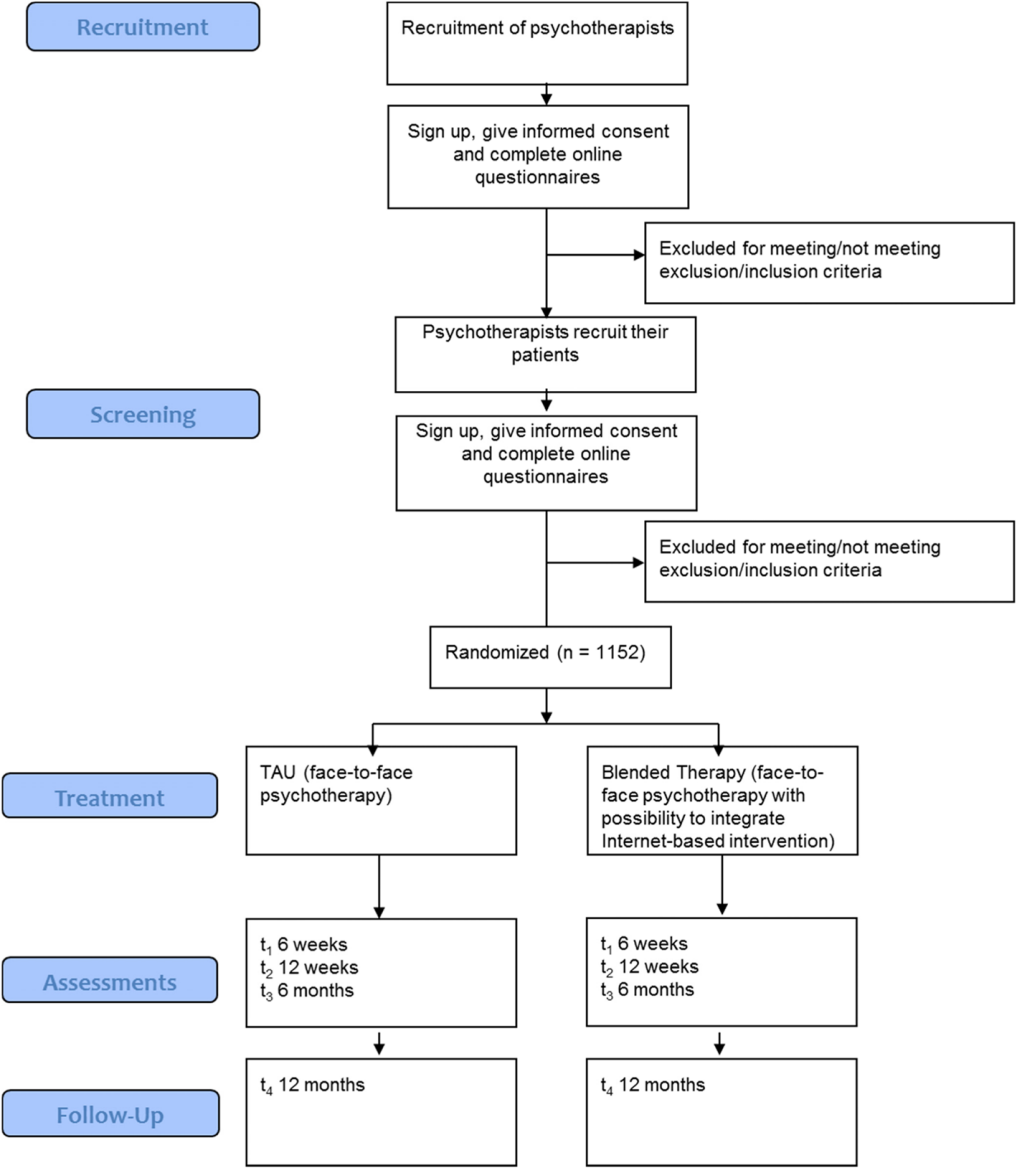
CONSORT flow diagram

Figure 1 depicts a CONSORT flow diagram. We will direct psychotherapists interested in participating to a study website that provides further information on the trial. Therapists willing to participate can sign up for participation and give electronic written informed consent. Once registered, therapists recruit participants within their pool of patients who are looking to start therapy. If patients are interested, they can sign up on the platform via a personalized link or code that they receive from their psychotherapist. Once patients have entered the platform, they will give electronic written informed consent, complete questionnaires, and are randomized to one of two groups: blended therapy (face-to-face therapy plus TONI) or face-to-face therapy.

#### Sample size {14}

The sample size calculation is based on a clinically relevant additive effect with an effect size of *d* = 0.2 in favor of blended therapy [20, 21]. To detect this effect in a randomized controlled trial with a t-test (alpha error level = 5%, two-sided) for independent samples at 6 months post-randomization with a power of 80 percent requires 400 subjects per treatment group. Randomization occurs at the patient level, but patients are simultaneously nested in therapists. With an average of 25 new treatment cases per psychotherapist per year [22], we conservatively assume that an estimated minimum of 5 patients per psychotherapist agree to participate in the trial. To adequately address nesting, the therapist effect must be considered. Based on recent studies, we assume an intraclass correlation of 0.05 [23]. This results in a design effect of 1.2 [design effect = 1 + (*n*_*C*_ − 1) × ICC = 1 + (5 − 1) × 0.05 = 1.20]. Additionally, we estimate that 20% of patients will drop out of the study by the time of the post-treatment assessment [24]. Considering these factors results in a required sample of at least (400 × design effect × missing values = 400 × 1.2 × 1.2) 1152 patients (~ 231 psychotherapists).

#### Recruitment {15}

Psychotherapists will be recruited through their chambers and professional associations via e-mail newsletters, advertisements in print distributions, and events. Participating psychotherapists will receive study material for their patients.

### Assignment of interventions: allocation

#### Sequence generation {16a}

A randomization mechanism is hard coded into the online platform that hosts the intervention prior to starting recruitment.

#### Concealment mechanism {16b}

After patients complete their baseline assessment, the platform automatically randomizes them to one of the two groups.

#### Implementation {16c}

Randomization will occur at the patient level. Once patients consent to participate and provide baseline data, they are randomized to blended therapy or face-to-face therapy in a 1:1 ratio. We expect therapists to contribute at least 5 patients each. To ensure that each therapist treats patients of both groups, we will use block randomization with a block size of four to randomize patients. The randomization will be automated after the baseline assessment is completed and carried out by the platform that hosts TONI.

### Assignment of interventions: blinding

#### Who will be blinded? {17a}

Due to the nature of psychotherapeutic treatment trials, neither participants nor therapists can be blind to treatment allocation. Therefore, group allocation will be masked for the statistical analyses concerning group differences between blended and face-to-face therapy. As data on usability and usage of the intervention is only available for the blended therapy group, these measures will not be included in the masked data set. A separate data set will contain these data and will be analyzed once the analysis of group differences is finalized.

#### Procedure for unblinding if needed {17b}

The design is open-label so unblinding for study coordinators will not occur.

### Data collection and management

#### Plans for assessment and collection of outcomes {18a}

##### Primary outcome

The primary outcome measure is mental distress, as measured by the composite score of the 7-item Generalized Anxiety Screener (GAD-7) and 8-item Patient Health Questionnaire (PHQ-8). Individually, both scales have demonstrated satisfactory validity and reliability and are widely used outcome measures in psychotherapy research [25–28]. As a composite score, the PHQ-ADS, a sum score of the GAD-7 and PHQ-9, has been found to have good convergent and construct validity as well as sensitivity to change in both anxiety and depression [29]. We do not expect deviations by using the PHQ-8 instead of the PHQ-9, since the PHQ-9 and PHQ-8 demonstrate equal diagnostic accuracy [30]. The PHQ-8 omits one of the PHQ-9’s items that assess thoughts about death or self-harm. Psychotherapists routinely monitor suicide ideation over the course of treatment. Since psychotherapists can only access their patient’s answers to the questionnaires when their patients are randomized to the blended therapy condition and we cannot ensure that psychotherapists review and follow up on their patients’ answers to questionnaires, we chose the PHQ-8 instead of the PHQ-9.

##### Secondary outcome measures

All secondary outcome measures (see Table 2) have shown satisfactory reliability and validity in previous studies. We will assess satisfaction with life with the 5-item Satisfaction with Life Scale (SWLS) [31, 32]. Physical and psychosocial functioning will be assessed with the 35-item Assessment of Quality of Life (AQoL-8D) [33]. For transdiagnostic symptoms, we will assess issues relating to eating/body image with the 8-item EDE-Q8 [34], alcohol and cannabis use with the 3-item AUDIT-C [35] and 4-item DUDIT-C [36], and sexual functioning with the 5-item Massachusetts General Hospital Sexual Functioning Questionnaire (MGH) [37]. Personality traits will be assessed with a brief version of the Personality Inventory for DSM-5 (PID5BF +) [38] and personality structure with the OPD-Structure Questionnaire Short form [39]. We will assess satisfaction with treatment with an adapted version of the 8-item Client Satisfaction Questionnaire (CSQ-8) [40, 41] and negative effects with the 20-item Negative Effects Questionnaire (NEQ) rated both by patients as well as psychotherapists [42]. Patients will also report their health care utilization with selected items of the FIMPsy [43]. Psychotherapists will rate symptoms and changes in general symptomatology using the CGI-S and CGI-C [44]. Patients and psychotherapists will rate the usability of the blended intervention with the 10-item System Usability Scale (SUS) [45]. At *t*_3_, psychotherapists will be asked to quantify the time they spent working with the online modules per month. They will also report in open-ended questions which potentials and limitations they see in applying blended care and how TONI can be improved.

##### Other measures

We will assess traumatization in childhood with the adverse childhood experiences questionnaire (ACE). Furthermore, we will assess self-efficacy with the 3-item General Self-efficacy Short Scale (ASKU) [46] and mental health self-efficacy with the 6-item Mental Health Self-Efficacy Scale (MHSES) [47]. Empowerment will be assessed with 2 open-ended questions. Patients will also rate their therapeutic agency with the 15-item Therapeutic Agency Inventory (TAI) [48]. Furthermore, they will rate their therapist’s activity and techniques with the 20-item Comparative Psychotherapy Process Scale (CPPS) [49] and facilitative skills with the 7-item common factor subscale of the Multitheoretical List of Therapeutic Interventions (MULTI) [50]. Patients and psychotherapists will rate therapeutic alliance with the 12-item Working Alliance Inventory (WAI-SR) [51]. Finally, three self-drafted items will assess expected treatment effects and the expected likelihood of dropping out at the beginning of therapy. At 6 months, patients will then also rate to what extent their symptoms changed and to what extent therapy helped them achieve these changes. For every face-to-face session, patients who receive blended therapy will report to what extent TONI was integrated into the face-to-face session and—if their psychotherapist activated this functionality—complete two rating scales, the Session Rating Scale (SRS) [52] and Outcome Rating Scale (ORS) [53]. To investigate therapist effects and patient-therapist matches, we will further assess therapist personality via the BFI-10 [54]. Patients and therapists will also rate their perceived similarity using a self-formulated item. If a psychotherapist signals that face-to-face therapy has terminated, two self-formulated items assess whether this termination can be considered a dropout and what the reasons for terminating therapy were.

#### Assessment of significant others

We will assess significant others at the beginning of treatment and at *t*_3_. Assessments of significant others will explore their expectations of treatment effects and dropout of the patient (3 self-drafted items), the perceived distress of the patient (GAD-7 and PHQ-8), their own distress (GAD-7 and PHQ-8), the consequences of their caregiving of the patient with the 31-item Involvement Evaluation Questionnaire (IEQ) [55], and perceived negative effects of treatment for the patient (NEQ). At the end of treatment, we will also ask them to rate the therapy-associated changes in terms of symptoms and their relationship (6 adapted items plus 3 open-ended questions).

#### Qualitative interviews

Ten strategically and sequentially selected psychotherapists will be invited to an in-depth semi-structured qualitative telephone interview about their experience and satisfaction with the treatment as well as perceived opportunities and limitations of the blended therapy approach. A strategic selection is applied to ensure a balanced representation of different therapeutic approaches and demographics.

Twenty strategically and sequentially selected patients will also be answering questions in an in-depth semi-structured qualitative telephone interview about their experience and satisfaction with the treatment as well as perceived opportunities and limitations of the blended therapy approach. A strategic selection is applied to ensure a balanced representation of different responder statuses, symptom severity, and demographics.

#### Plans to promote participant retention and complete follow-up {18b}

Participants will receive e-mail notifications that questionnaires are available on the TONI platform. Questionnaires will be available for 4 weeks, and patients will be reminded weekly as long as they do not complete the assessment. Participants who end or drop out from psychotherapy will still be invited to participate in the assessments. Patients and psychotherapists will receive a monetary incentive to complete assessments. Patients will receive €100 total for completing all four major assessments. Psychotherapists will receive €100 total for the completion of assessments per patient.

#### Data management {19}

Except for the qualitative interviews, all data is gathered online and will be recorded automatically. Data will be stored on a secure server. After study completion, de-identified data will be made publicly available in an Open Science Framework (OSF) repository.

#### Confidentiality {27}

Participants will register on the TONI platform with their e-mail addresses to receive reminders and recover their accounts. All data is stored on a secure platform, and personal data will be masked in the databases.

#### Plans for collection, laboratory evaluation, and storage of biological specimens for genetic or molecular analysis in this trial/future use {33}

n/a: No biological specimens will be collected.

## Statistical methods

### Statistical methods for primary and secondary outcomes {20a}

For the primary analyses, we will include data of all randomized patients (intention-to-treat (ITT) analysis). To account for the nesting of patients (level 1) in psychotherapists (level 2), the ITT effect is estimated 6 months after the start of treatment using multilevel models. In an exploratory analysis, the influence of the actual use of the online modules is analyzed. For this purpose, the extent of actual use (e.g., number of modules used) and the time of use are inserted as moderators, and conditional effects are estimated. The same analyses are repeated for the secondary outcome measures. An appropriate alpha error correction is applied to avoid alpha error accumulation for secondary outcomes (Bonferroni-Holm). Effect sizes for pre- to post-treatment and between-group comparisons for the main and secondary outcomes will be calculated to evaluate the effectiveness of the intervention. The qualitative interviews will be recorded, transcribed, and analyzed according to a qualitative content analysis by trained staff with an appropriate review of the assessment quality (inter-rater reliability).

### Interim analyses {21b}

No interim analysis is planned in this trial.

### Methods for additional analyses (e.g., subgroup analyses) {20b}

Moderator and mediator analyses will be conducted at a later stage (subsequent to the main outcome paper) to analyze whether certain factors predict the differential effects of the interventions or mediate treatment effects.

### Methods in analysis to handle protocol non-adherence and any statistical methods to handle missing data {20c}

We will estimate the ITT effect, which is the effect of treatment assignment. This allows us to evaluate the effect of such an assignment (regardless of whether it is followed or not). For the primary analyses, missing values are multiply imputed or estimated using full information maximum likelihood, depending on the variables for which missing values occur. Both procedures are considered gold standards in dealing with missing values for which the missing at random (MAR) assumption applies. Additional covariates are considered to make the MAR assumption plausible and increase the precision of the effect estimates.

### Plans to give access to the full protocol, participant-level data, and statistical code {31c}

De-identified participant-level data as well as analysis code will be made publicly available through a research repository (OSF).

### Oversight and monitoring

#### Composition of the coordinating center and trial steering committee {5d}

There is no independent Trial Steering Committee. Instead, the research team reviews recruitment rates weekly and meets on a monthly basis to discuss key milestones as well as the conduct the research, ensuring the integrity of the protocol and conduct of the study. Any significant amendments to the study protocol will be provided to and approved by the ethics committee of Psychologische Hochschule Berlin before implementation.

#### Composition of the data monitoring committee, its role, and reporting structure {21a}

A data monitoring committee is not planned for this trial. Instead, all patients are enrolled in psychotherapy in routine care, all data will be coded directly to the system where no adjustments can be made to data, and all actions are logged.

#### Adverse event reporting and harms {22}

During the trial, all patients are enrolled in outpatient therapy in routine care. In case of adverse events, psychotherapists will attend to the adverse events and take appropriate measures to alleviate them. At post-assessment, we will collect the negative effects caused by the treatment systematically, as suggested by Rozental and colleagues [56]. Participants — psychotherapists, patients, and their significant other — will complete the NEQ and are encouraged to further note all adverse events that occurred during treatment in an open-ended question. In addition, other markers indicative of adverse events, like hospitalization, as assessed by FIMPsy, and deterioration rates, will be published.

#### Frequency and plans for auditing trial conduct {23}

Psychologische Hochschule Berlin does not have a dedicated research audit department. The research team meets monthly to review trial conduct.

#### Plans for communicating important protocol amendments to relevant parties (e.g., trial participants, ethical committees) {25}

Any amendments to the study protocol would have to be approved first by Psychologische Hochschule Berlin’s ethics committee. If approved, these changes would have to be reported in the trial register. Finally, any amendments would be reported in the trial paper.

#### Dissemination plans {31a}

The trial results will be published in a peer-reviewed journal. The results will also be presented at scientific conferences. A popular science summary of the results will be posted online for laymen and study participants. An anonymized data set as well as statistical code used to analyze the data will be published in a data repository on OSF.

## Discussion

This is the first RCT to investigate a transdiagnostic and transtherapeutical as well as modular blended intervention in German psychotherapy in routine care. Unique to this intervention, TONI will be the first Internet-based intervention to cover therapeutic principles across cognitive -behavioral, psychodynamic, and systemic therapy. The majority of transdiagnostic Internet-based interventions focus on anxiety and depression. However, this focus neglects other issues of the internalizing and the whole externalizing spectrum of pathology. TONI taps into this, with modules corresponding to transdiagnostic processes and principles across the whole spectrum of mental health. If the blended therapy’s effectiveness and utility are demonstrated, TONI can be a useful and promising tool for psychotherapists with different theoretical backgrounds and for a wide range of patients. The modular and flexible structure of TONI allows psychotherapists to tailor the blended treatment to their and their patients’ needs, making it suitable for real-life settings.

Our results will highlight whether integrating digital tools into psychotherapy has a positive impact. Importantly, this trial will be sufficiently powered to detect small differences between blended and face-to-face therapy to better understand blended therapy’s potential in terms of effectiveness. To be reflective of the broad inclusion criteria, transdiagnostic spectra and symptoms will be included to capture changes beyond anxiety and depression. Besides effectiveness rates, other markers are important determinants of interventions’ feasibility and dissemination potential. We will cover this broadly, assessing satisfaction with treatment and negative effects and qualitatively interviewing patients and psychotherapists. Our results will shed light on how psychotherapists and patients use digital tools and the advantages and disadvantages of such an integration. Notably — since there is a lack of research on how Internet-based interventions and psychotherapy should be blended — we will investigate whether the degree of integration of online interventions in psychotherapy matters and to what extent. In addition, our findings will contribute to knowledge on which patients benefit from blended therapy and which underlying mechanisms mediate effects.

The trial has several limitations: This study compares two active treatments, hypothesizing that blended therapy may be superior to regular psychotherapy in routine care. One of the assumptions behind this hypothesis is that patients may receive a higher treatment dosage by using additional online modules. However, the treatment dosage is dependent on whether psychotherapists actively engage with the intervention and assign modules. A strength of this is that it represents real-world behavior and as such is externally more valid. However, it may limit conclusions about the actual effect of making use of the full potential of the modules. We collect data on the usage of the modules and will add it as moderators to evaluate its possible impact on the results. Psychotherapists will also be in charge of recruiting their patients. This may constitute a bottleneck for the recruitment of patients, potentially jeopardizing recruitment rates. The recruitment procedure may limit the generalizability of the results, as psychotherapists choose which patients to invite to participate in the study. In addition, we are not conducting a cost analysis.

The present RCT investigates the superiority of blended over psychotherapy alone in routine care. The integration of online interventions like TONI that use a common therapeutic language and address therapeutic principles shared across therapeutic approaches has the potential to advance the effectiveness of psychotherapy. TONI may not only improve the effectiveness of psychotherapy in routine care but help save therapists’ resources and close treatment gaps.

## Trial status

Psychotherapist and patient recruitment commenced in mid-June 2022 and is expected to be completed by June 2023.

## Data Availability

After the publication of the trial results, a de-identified data set as well as analysis code will be made publicly available on the research data repository OSF.

## Abbreviations

ACE: Adverse Childhood Experiences Questionnaire
AQoL-8D: Assessment of Quality of Life
ASKU: General Self-efficacy Short Scale (Allgemeine Selbstwirksamkeit Kurzskala)
AUDIT-C: Alcohol Use Disorders Identification Test-Concise
DUDIT: Drug Use Disorder Identification Test
CBT: Cognitive behavioral therapy
CGI-C: Clinical Global Impression-Change
CGI-S: Clinical Global Impression-Scale
CONSORT: Consolidated Standards of Reporting Trials
CPPS: Comparative Psychotherapy Process Scale
CSQ-8: Client Satisfaction Questionnaire
DRKS: Deutsches Register Klinischer Studien (German Register of Clinical Trials)
EDE-Q8: Eating Disorder Examination-Questionnaire 8
FIMpsy: Questionnaire of Medical and Non-Medical Health Care Utalization by Mental Disorders (Fragebogen zur Inanspruchnahme medizinischer und nicht medizinischer Versorgungsleistungen bei psychischen Erkrankungen)
GAD-7: Generalized Anxiety Screener 7
GBA: Gemeinsamer Bundesausschuss
IEQ: Involvement Evaluation Questionnaire
ITT: Intention-to-treat
MAR: Missing at random
MGH: Massachusetts General Hospital Sexual Functioning Questionnaire
MHSES: Mental Health Self Efficacy-Scale
MULTI: Multitheoretical List of Therapeutic Interventions
NEQ: Negative Effects Questionnaire
OPD: Operationalized Psychodynamic Diagnostic (Operationalisierte Psychodynamische Diagnostik)
ORS: Outcome Rating Scale
OSF: Open Science Framework
PHQ-ADS: Patient Health Questionnaire Anxiety and Depression Scale
PHQ-4/8/9: (4/8/9-item) Patient Health Questionnaire
PID-5-BF +: Personality Inventory for DSM-5 Brief Form
PsyTOM: Psychotherapy through blended therapy with transdiagnostic online modules
RCT: Randomized controlled trial
SPIRIT: Standard Protocol Items: Recommendations for Interventional Trials
SRS: Session Rating Scale
SUS: System Usability Scale
SWLS: Satisfaction with Life Scale
TAI: Therapeutic Agency Inventory
TONI: Therapeutic online intervention
WAI-SR: Working Alliance Inventory-Short Revised
